# Side‐of‐onset of Parkinson's disease in relation to neuropsychological measures

**DOI:** 10.1002/brb3.590

**Published:** 2016-10-19

**Authors:** Edward J. Modestino, Chioma Amenechi, AnnaMarie Reinhofer, Patrick O'Toole

**Affiliations:** ^1^ Department of NeurologyBoston University School of MedicineBostonMAUSA

**Keywords:** cognitive, neuropsychology, Parkinson's disease, Side‐of‐onset

## Abstract

**Background:**

Parkinson's disease (PD) usually emerges with a unilateral side‐of‐onset (left‐onset: LOPD; right‐onset: ROPD; Marinus & van Hilten, 2015) due to an asymmetrical degeneration of striatal dopaminergic neurons (Donnemiller et al., *Brain*, 135, 2012, 3348). This has led to a body of research exploring the cognitive, neuropsychological, and clinical differences between LOPD and ROPD (e.g., Verreyt et al., *Neuropsychology Review*, 21, 2011, 405).

**Methods:**

Thirty ROPD and 14 LOPD cases were drawn from a Boston clinic specializing in PD. Various cognitive and neuropsychological measures were used in an attempt to discover if there were indeed any differences between LOPD and ROPD in this cohort.

**Results:**

For LOPD, duration of illness was found to be significantly greater than that of ROPD. However, further testing was able to confirm that despite this difference, it was not the cause of the other significant differences found. Furthermore, this increased duration was consistent with a previous study (Munhoz et al., *Parkinsonism and Related Disorders*, 19, 2013, 77). Performance on the Digit Span Backward (DSB) was found to be significantly poorer in LOPD than ROPD, suggesting compromised executive function in LOPD. Additionally, LOPD had significantly greater anxiety on the DASS Anxiety scales than ROPD. However, unlike Foster et al (*Cognitive and Behavioral Neurology*, 23, 2010, 4), this increased anxiety could not account for the poorer performance on the DSB for LOPD. Finally, ROPD had significantly greater magical ideation than LOPD, which can be explained by the theory put forth by Brugger and Graves (*European Archives of Psychiatry*, 247, 1997, 55).

**Conclusion:**

Clear and significant differences between LOPD and ROPD were found within our cohort. LOPD showed greater impairment of working memory, greater anxiety, and greater duration of illness—all independent of one another; whereas, those with ROPD had greater magical ideation, also independent of any other variables.

## 1 Introduction

Initially, when patients are diagnosed with Parkinson's disease (PD), they often report that motor symptoms appear unilaterally (Marinus & van Hilten, 2015). Although PD eventually progresses to bilateral symptoms, the initial side‐of‐onset (LOPD: left‐onset PD, ROPD: right‐onset PD) may still show more severe symptoms throughout disease progression (Marinus & van Hilten, 2015). This is due to greater degeneration of dopaminergic neurons in the dorsal striatum of the brain hemisphere contralateral to the body's side‐of‐onset (Donnemiller et al., 2012). This asymmetric depletion leads to further dysfunction of the neural circuits connected to the basal ganglia, which influence cognitive abilities (Verreyt et al., 2011). Additionally, it has been reported that brain asymmetry may have a role in emotion and/or motivation (Fetterman, Ode, & Robinson, 2013). Based on this asymmetrical degeneration, much research has focused on the cognitive/neuropsychological and even clinical differences between LOPD and ROPD (e.g., Verreyt et al., 2011). However, recent research has suggested that the cognitive differences are not seen in the recently diagnosed, unmedicated, early stage of PD, such as those in Hoehn and Yahr (H&Y) Stage 1 (Erro et al., 2013; Pellicano et al., 2015). In the conclusion of one of these studies, it was suggested that reported cognitive differences between LOPD and ROPD in previous studies may in fact be due to medication treatment (Pellicano et al., 2015). However, this is counterintuitive and questionable considering that all patients, both LOPD and ROPD, have been on treatment prior to and/or during studies showing these differences. In other words, the most notable, if not the only, difference between groups in these studies should be side‐of‐onset. Poletti et al. (2013) has suggested an alternative explanation that with greater progression of PD, cognitive differences between LOPD and ROPD may become more obvious. The proceeding is a review of studies that, overwhelmingly, suggest these differences are indeed authentic.

ROPD has been associated with cognitive/neuropsychological and clinical differences when compared with LOPD and/or controls. Verreyt et al. (2011) provided a review which summarized the deficits in ROPD related to tasks involving language and verbal memory. Cheesman et al. (2005) also found this verbal memory deficit in ROPD. Additionally, verbal creativity deficits have been seen in ROPD (Drago, Foster, Skidmore, & Heilman, 2009). Those with ROPD show a deficit in mental rotation related to one's self‐view (Bowen, Burns, Brady, & Yahr, 1976; Cronin‐Golomb, 2010). In the clinical domain, those with ROPD have shown differences such as greater apathy (Bogdanova & Cronin‐Golomb, 2012), more severe psychosis (Cubo, 2010), and longer duration of illness associated with greater anxiety and depressive symptoms (Foster et al., 2011).

Similarly, LOPD has also been associated with cognitive/neuropsychological and clinical differences when compared with ROPD and/or controls. Verreyt et al. (2011) imparted deficits in spatial attention, visuospatial orienting and memory, and mental imagery in LOPD. Consistent with these visual deficits in LOPD, deficits in visuospatial ability/attention (Norton, Jaywant, Gallart‐Palau, & Cronin‐Golomb, 2015; Poletti et al., 2013) and spatial planning (Cheesman et al., 2005) have been found. Interestingly, a deficit in spatial memory was shown to be associated with smaller substantia nigra volume (using MRI) in LOPD (Foster, Black, Antenor‐Dorsey, Perlmutter, & Hershey, 2008; Those with LOPD have shown significant impairment of working memory on the Digit Span Backward (DSB) task, which was associated with significantly greater depression in one study (Foster et al., 2013) and significantly greater anxiety in another study (Foster et al., 2010). Furthermore, those with LOPD show a deficit in mental rotation related to object‐view (Cronin‐Golomb, 2010; Lee, Harris, & Calvert, 1998). With regard to pragmatics (meaning), those with LOPD have shown a deficit in language fluency, using fewer verbs, and constructing shorter sentences (Holtgraves, McNamara, Cappaert, & Durso, 2010). Additionally, deficits in prosodic emotional recognition/emotional tone of voice (Ventura et al., 2012) and religiosity (Butler, McNamara, & Durso, 2011; Giaquinto & Bruti, 2011) have been seen in LOPD. In the clinical domain, those with LOPD have shown differences in greater duration of illness (Munhoz et al., 2013), decreased self‐awareness of motor deficits (Maier et al., 2012), greater risk of dementia and REM sleep behavior disorder (Baumann, Held, Valko, Wienecke, & Waldvogel, 2014), and greater severity of depression (Dewey, Tajena, & McClintock, 2013).

## 2 Methods

### 2.1 Objectives

Based on the studies in the preceding review, it appears that there are indeed cognitive, neuropsychological, and clinical differences between LOPD and ROPD. As we had access to these populations ourselves, we decided to examine any and all differences between LOPD and ROPD. Based on previous research, we decided to use a range of cognitive and neuropsychological measures. We hypothesized that there would be a greater working memory deficit in LOPD, as shown using the DSB task, that might be associated with greater depression (Foster et al., 2013) and anxiety (Foster et al., 2010). Thus, we paired the use of the DSB with measures of depression and anxiety (DASS; see 2.3 for details). Based on the evidence of more severe psychosis in ROPD (Cubo, 2010), we also hypothesized increased schizotypy as shown in a measure of magical ideation (MIS; again, please refer to the 2.3 section for specifics on measures) in ROPD. Other than these hypotheses, we followed a data‐driven approach, free of the biases of strict hypothesis‐driven approaches. We were open to the possibility that there may be many cognitive, neuropsychological, and clinical differences between LOPD and ROPD, based on previous research findings. We wanted the data to tell us what was indeed there without automatically restricting hypotheses which could obscure the truth in the data. Furthermore, since we employed measures previously used in other studies, and since replication is the cornerstone of scientific research, we had hoped to add to the body of current literature, and perhaps find evidence consistent with previous research.

### 2.2 Participants: behavioral experiment

In the behavioral experiment, all participants were diagnosed with idiopathic Parkinson's disease by a board certified movement disorders specialist, and Director of Movement Disorders clinics at the Boston VA who recruited patients for the study from the Veteran's Administration Health System in Boston, MA, USA. Based on our recruitment at the VA, the majority were veterans.

This study was approved by the Institutional Review Board (IRB) of VA Boston Healthcare System in Jamaica Plain, Boston, MA, USA. All participants completed an informed consent as specified and approved by said IRB. A consecutive sampling method was employed which was based upon the time allotted for the study and the budget. Our patients were primarily recruited by their physician in the PD clinic, based on who came in. They were asked if they wished to participate. Those who followed up (self‐selecting) were scheduled, and paid $10 an hour for participation in the neuropsychological testing and $30 for returning the take‐home packet of inventories. Exclusionary criteria included dementia or severe cognitive impairment based on measures of mental status (refer to measures in 2.3). We made sure that both side‐of‐onset groups were equivalent in measures of mental status and age of onset of illness.

### 2.3 Procedures: neuropsychological testing

All participants were given a battery of neuropsychological tests to assess possible comorbid dementia and cognitive impairment while on medication. These included the Mini‐Mental State Examination (MMSE; Nazem et al., 2009), the Montreal Cognitive Assessment (MoCA; Nasreddine et al., 2005), the Wechsler Test of Adult Reading (WTAR; Holdnack, 2001), the Matrix Reasoning test which is a subtest within the Wechsler Adult Intelligence Scale (WAIS; Wechsler, 2008), Digit Span Backward (DSB; a subtest of the Wechsler Memory Scale–III; Wechsler, 1997), and the Stroop test (Stroop, 1935). Additionally, participants were assessed for mood function using the Depression, Anxiety, and Stress Scale (DASS; Henry & Crawford, 2005) and the Magical Ideation Scale (MIS; Eckblad & Chapman, 1983).

Levodopa equivalency dosages (LED), to be used in statistical analyses, were calculated using standardized formulae to compare dosing levels across the variety of dopamine replacement therapies that our participants with PD were taking (Tomlinson et al., 2010). All testing reported in this paper was completed on medication.

### 2.4 Behavioral data processing and statistical analysis

Simple analysis of means was completed within Excel. Additionally, data were exported from Excel for hypothesis testing in IBM SPSS.

We employed multivariate mixed‐effects linear regression analyses to test for associations between the side‐of‐onset (dependent variable) and neuropsychological and clinical measures (independent variables). All models were adjusted for age, education, sex, and handedness. All significant findings were further teased apart in additional regressions to account for the true nature of interactions between all significant variables, which was done by switching independent and dependent variables. We allowed for outcome‐specific fixed effects and subject‐specific and measure‐specific random effects. These multivariate analyses are more realistic models of the outcomes than using independent regression models for each outcome. Since all information within each subject is utilized, we are able to provide more interpretable and consistent results than simpler statistical models. Moreover, the problem of multiple comparisons is removed when viewed from these models (Gelman, Hill, & Yajima, 2012). These multivariate models provide higher power for detecting small but clinically important differences compared to independent regression models for each outcome (Goldstein, 2010). These analyses were performed using IBM SPSS Statistics (version 22, IBM, Armonk, NY, USA). It was clear that choosing this specific analysis would divulge the true nature and interactions between all of these variables.

## 3 Results

In the behavioral experiment, the participants included patients (*n* = 44; 41 males, three females) diagnosed with idiopathic Parkinson's disease. The mean age was 68.818 years within a range of 42:89 years. Side‐of‐onset included 14 LOPD (one female) and 30 ROPD (two females). Race consisted of 40 Caucasians, two African‐Americans, and one Native‐American. The majority of the participants were right handed (*n* = 29), some were left handed (*n* = 6), and some ambidextrous (*n* = 9). All but two (42 of the 44; 95.45%) were high school graduates. Thirty‐one (70.45%) had some college experience or an associate's degree. Twenty‐one (47.72%) had bachelor's degree. Nine (20.45%) had postgraduate degrees or at least 1 year of postbaccalaureate education. Years of education ranged from 4 to 22 years, with the mean at 14.96 years. The overall Hoehn and Yahr scale score median was 2 (Q1: 2, Q2: 2, Q3: 3, Q4: 4; IQR: 1) within a range of 1:4, with the majority being H&Y stage 2 (*n* = 21), stage 1 (*n* = 2), stage 3 (*n* = 17), and stage 4 (*n* = 4). Thus, nearly 86% (86.36%, *n* = 38) were in stages 2–3. Duration of PD illness had a mean across the group of 6.375 years within a range of 1:20 years. Refer to Table 1 for details.

**Table 1:** Demographics of PD participants

Participants	*N* = 44		41 males		3 females	
Handedness	Right: 29		Left: 6		Ambidextrous: 9	
Age	Range: 42:89 years				Mean: 68.818 years	
Education	Range: 4:22 years				Mean: 14.96 years	
side‐of‐onset	LOPD: 14 (1 female)				ROPD: 30 (2 females)	
Duration of PD	Range: 1:20 years				Mean: 6.375 years	
H&Y	Q1: 2	Q2: 2	Q3: 3	Q4: 4	IQR: 1	Median: 2

LOPD, left‐onset Parkinson's disease; ROPD, right‐onset Parkinson's disease; H&Y, Hoehn and Yahr stage of Parkinson's disease; Q1:Q4, quartile values; IQR, Interquartile range (Q3‐Q1).

All results were obtained using multivariate mixed‐effects linear regression analysis (*R* ^2^ = .935) without the need to correct for multiple comparisons. Positive *t* values indicate significantly greater for ROPD; whereas, negative *t* values indicate significantly greater for LOPD. There were no significant effects of age, gender (sex), race, handedness, education, age of disease onset, levodopa equivalency dosages (LED), mood, or mental status as measured on the MMSE and MoCA on the dependent variable of side‐of‐onset (LOPD and ROPD). However, there was a significant effect (*t*_35_ = −3.384, *p* = .007) of duration of illness for LOPD (mean: 6.5714 years with a range of 1:20 years) greater than ROPD (mean: 6.28 years with a range of 1:18 years). There was a significant (*t*_35_ = 3.886, *p* = .003) difference in the DSB such that those with ROPD (mean: 6.3 with a range of 2:11) performed significantly greater than those with LOPD (mean: 5.9283 with a range of 4:8). Also, there was a significant (*t*_35_ = 2.485, *p* = .032) difference in the MIS such that those with ROPD (mean: 5.8421, range of 0:13) scored significantly higher in magical ideation than those with LOPD (mean 2.6363, range of 0:10). Finally, there was a significant (*t*_35_ = −4.843, *p* = .001) difference in the DASS Anxiety scale such that those with LOPD (mean 2.5, range of 1:4) suffered from significantly more anxiety than those with ROPD (mean 2.125, range of 1:5). A regression analysis collapsing across LOPD and ROPD, and using duration of illness as the dependent variable, with independent variables of MIS, DSB, and DASS anxiety failed to reach significance. Thus, the significant difference in the duration of illness between LOPD and ROPD cannot account for the differences between groups on the Magical Ideation Scale, the Digit Span Backward, or the DASS Anxiety. A regression analysis on LOPD data using DASS Anxiety as the dependent variable with Digit Span Backward as the independent variable failed to reach significance. Refer to Table 2 for a summary of these significant results.

**Table 2:** Significant differences between sides of onset (dependent variable)

Test (independent variables)	*p* value	*t* value	*df*	LOPD X—	ROPD X—
Duration	.007	−3.384	35	6.5714	6.28
DSB	.003	3.886	35	5.9283	6.3
MIS	.032	2.485	35	2.6363	5.8421
DASS	.001	−4.843	35	2.5	2.125

LOPD, left‐onset Parkinson's disease; ROPD, right‐onset Parkinson's disease; DSB, Digit Span Backward; MIS, Magical Ideation Scale; DASS, DASS Anxiety Scale.

All results were obtained using multivariate mixed‐effects linear regression analysis (*R* ^2^ = .935) without the need to correct for multiple comparisons. Positive *t* values indicate significantly greater for ROPD; whereas, negative *t* values indicate significantly greater for LOPD.

## 4 Discussion

We found duration of illness to be significantly greater in LOPD than ROPD within our sample. This is consistent with earlier findings that duration of illness has been shown to be greater in those with LOPD than ROPD in a group of those with a duration of illness longer than 20 years (Munhoz et al., 2013). Importantly, as we found a significant difference in duration as an independent variable within the regression model (dependent variable was side‐of‐onset), with LOPD significantly greater than ROPD, we were concerned that this difference between groups may have accounted for all the differences we saw between groups. However, collapsing across LOPD and ROPD and using disease duration as the dependent variable resulted in nothing of significance in any of the other measures. If any true differences in measures were simply due to duration of illness between groups, and not the groups themselves, they would have been revealed using this analysis. Thus, despite the significant difference in illness duration between LOPD and ROPD groups, the other significant differences that we found in other measures cannot be explained by this duration of illness. Interestingly, this same significant difference in duration of illness for LOPD (vs. ROPD) has been found in a previous study (Munhoz et al., 2013).

We found that there was a significant difference in performance of the Digit Span Backward, with LOPD scoring significantly poorer than ROPD. The Digit Span Backward is a subtest of the Wechsler Memory Scale–III (Wechsler, 1997). The Digit Span Forward is primarily a measure of short‐term attention; whereas, the Digit Span Backward expands on this to include working memory (Acton, 2013). Therefore, our results appear to indicate a significant difficulty in working memory for those with LOPD versus ROPD. This is consistent with previous research showing that those with LOPD have a greater cognitive decline as shown in various tests of executive functions**,** presumably due to greater degeneration of the right hemisphere dopamine system (Tomer, Levin, & Weiner, 1993). Foster et al. (2013) found this same decline in the Digit Span Backward in LOPD, however, it was limited to those with LOPD and comorbid depression. Importantly, our measure of depression from the DASS did not show anything of significance in relation to any of other measures, including the Digit Span Backward, in the model. Interestingly, in our study, LOPD experienced significantly more anxiety, as shown on the DASS anxiety measure, than those with ROPD. Foster et al. (2010) found that this greater anxiety in LOPD was associated with worse performance on the Digit Span Backward. Therefore, we considered the possibility that heightened anxiety seen in our sample of LOPD was associated with the decline in working memory as shown with poor performance on the Digit Span Backward task. However, a regression using anxiety scores among those with LOPD as the dependent variable (from DASS anxiety) failed to result in a significant covariance (either positive or negative) with the working memory scores from the Digit Span Backward. Thus, it appears that for this study, DASS anxiety scores did not show a significant relationship with working memory scores. It is unclear that anxiety played a role in poorer performance on a working memory task for LOPD in this study. Therefore, the greater anxiety seen in LOPD than ROPD may not have affected working memory in LOPD. However, there was still significantly more anxiety in LOPD than ROPD in this study. It could be the difference in our measure of anxiety versus that of Foster et al. (2010) that accounts for the differences in findings. The two (DASS anxiety measure for this study, and State‐Trait Anxiety Inventory for Foster et al., 2010) may not be measuring the exact same thing. Thus, anxiety may account for the working memory deficit we saw in LOPD in our study, even though higher anxiety (in our measure) we see in our LOPD did not covary with the DSB in a negative relationship. Alternatively, right hemispheric dopaminergic degeneration of the basal ganglia (specifically the striate) may somehow be affecting the right limbic system (including amygdala) connections to the prefrontal cortex that may account for higher anxiety in LOPD. However, there is merely speculation at this point.** **Foster et al. (2011) reported that duration of illness was positively associated with severity of anxiety and depressive symptoms in ROPD. However, in our study, greater anxiety was seen in LOPD, and duration as a dependent variable failed to reveal any significant covariance with any measure including DASS anxiety.

Finally, we found a significantly greater score in magical ideation on the MIS in ROPD than LOPD. As LED was used as an independent variable in the model and no significant effect was seen in LED between side‐of‐onset groups (dependent variable) for LED, this cannot explain the differences seen in magical ideation between groups. Brugger and Graves (1997) found right hemispatial neglect‐like inattentional behavior to be paired with increased scores on the MIS (a measure of schizotypy) in neurotypicals. They attributed this to be due to a left hemispheric hypodopaminergic state (relative to the right hemisphere) that led to the attentional bias (right inattention), but separately to a disinhibition of right hemispheric semantic regions. They further expounded that this disinhibition could lead to the magical thinking and possibly delusions. ROPD is associated with greater hypodopaminergic state in the left hemisphere leading to the initial motor symptoms in the right side of the body (Donnemiller et al., 2012). Thus, the assumption would be that in our ROPD (13 males and one female) with greater dopaminergic degeneration in the left hemisphere, there would also be higher MIS scores. This is in fact what we found. Furthermore, this is consistent with the literature suggesting more severe psychosis in ROPD (Cubo, 2010).

There were some notable limitations in this study. Based on the fact that this study was run out of and recruited from a VA hospital, a much larger sample were males. Furthermore, the discrepancy in laterality (side‐of‐onset), in addition to gender, was seen in this specific clinic. Far more patients in this specific clinic were ROPD than LOPD. The LOPD tends to be rare in this specific clinic. Thus, we simply used what we were able to obtain within our limited population. Future research with a more balanced gender and side‐of‐onset are needed to confirm these results.

## 5 Conclusion

In our cohort, the duration of illness was significantly greater for LOPD that cannot explain any of our other significant findings. We found that LOPD performed significantly poorer on the DSB, suggesting compromised executive functioning and also had significantly greater anxiety independent of one another. Finally, those with ROPD showed significantly greater magical ideation.

## Conflict of Interests

None declared.
